# Hip arthroplasty fatality related to dabigatran induced gastrointestinal haemorrhage

**DOI:** 10.1308/003588414X13824511649779

**Published:** 2014-01

**Authors:** A Carter, P Sarda, M George, S Corbett

**Affiliations:** Guy’s and St Thomas’ NHS Foundation Trust,UK

**Keywords:** Anticoagulation, Dabigatran, Hip arthroplasty

## Abstract

We report a fatality due to massive gastrointestinal haemorrhage in a patient receiving prophylactic dabigatran etexilate following a total hip replacement. A 79-year-old woman was commenced on dabigatran for venous thromboembolic prophylaxis following a total hip replacement. She presented again four days after surgery with haematemesis and hypotension but her coagulopathy could not be corrected, leading to her death. This case highlights the lack of reversal agent for dabigatran etexilate that resulted in this fatal complication.

Dabigatran etexilate (Pradaxa®; Boehringer Ingelheim, Ingelheim am Rhein, Germany), a direct inhibitor of the enzyme thrombin, is marketed worldwide for primary prevention of venous thromboembolism (VTE) in patients undergoing total hip or knee replacement surgery. It has been shown to have comparable efficacy with low molecular weight heparin (LMWH) in phase III trials.^1^ It also showed patient and staff advantages of a higher level of acceptability and greater ease of administration. Dabigatran differs from LMWH in that there is currently no effective reversal agent. This has been highlighted by several recent case reports of fatality emerging from patients who were on dabigatran for other conditions such as atrial fibrillation.^2–5^

Following lower limb joint replacement, dabigatran is one of the drugs recommended by the National Institute for Health and Care Excellence (NICE) for VTE prophylaxis.^6^ Our institution followed this recommendation until recently. This case report aims to highlight significant gastrointestinal bleeding as a potential side effect of this drug, which, in the absence of a reversal agent, resulted in the death of a patient following an elective total hip replacement (THR).

## Case history

A 79-year-old woman underwent a routine right THR without any intraoperative complications. Her past medical history included a previous duodenal ulcer that had been asymptomatic for a few years prior to the surgery, gastrooesophageal reflux disease, hypertension, a coronary bypass, aortic stenosis, hypothyroidism and chronic obstructive pulmonary disease. She had undergone a THR on the other side previously, where dabigatran had been used for VTE prophylaxis without any side effects. Medications included clopidogrel (stopped ten days prior to surgery and not restarted after surgery while on dabigatran) in addition to suitable medication for other co-morbidities listed above. Her renal function tests revealed an estimated glomerular filtration rate (eGFR) of 65ml/min preoperatively.

The patient was started on dabigatran after surgery as per the hospital’s and NICE guidelines for VTE prophylaxis after THR. As she was over 75 years old, she received a reduced dosing regime consisting of a starting dose of 75mg 4 hours after surgery to be followed by 28 days of 150mg once a day.

The patient was discharged on day 3 following her surgery but readmitted on day 5 with abdominal pain and hypotensive shock following haematemesis. She was transferred to the intensive care unit. She received packed red blood cells, platelets, fresh frozen plasma, tranexamic acid and vitamin K, and had haemofiltration in an attempt to correct the coagulopathy. Repeated upper gastrointestinal endoscopies showed a generalised gastritis with superficial ulcerations. Adrenaline injections and clips were applied to bleeding areas. Although she had a history of duodenal ulcers, no active lesions were seen on endoscopy. Despite vigorous resuscitation, the treatment was unsuccessful and she died on day 9 after surgery. The postmortem report confirmed multiorgan failure secondary to shock from a massive gastrointestinal haemorrhage.

## Discussion

Dabigatran is a relatively new drug in the VTE market and has been recommended by NICE since 2008.^6^ Until the end of 2011, over 50,000 treatment courses (for all causes) were prescribed and 369 spontaneous adverse drug reactions in the UK have been reported to the Medicines and Healthcare products Regulatory Agency (MHRA).^7^ Out of these, 91 reports pertained to haemorrhagic reaction in patients treated for thromboprophylaxis with the gastrointestinal tract being the most common site (42 patients). Thirteen reports received in association with dabigatran had a fatal outcome, of which nine cases specified that the indication for use was thromboprophylaxis.

An review across Europe of haemorrhagic events in association with dabigatran resulted in updated monitoring information in patients with impaired renal function, highlighted in the MHRA drug safety bulletin in December 2011.^8^ The summary of product characteristics guidance highlights that there is limited clinical experience with regard to the elderly (those over 75 years old) and that these patients should be treated with caution.^9^ It also states that there is very limited clinical experience in patients with a body weight of under 50kg or over 110kg. It specifies that patients with moderate renal failure (eGFR 30–50ml/min) should receive the reduced dose of 150mg per day and that dabigatran is contraindicated in those with severe renal impairment (eGFR under 30ml/min).

In our case, there was no error in the prescription, which adhered both to NICE guidelines and manufacturer recommendations. Even though the patient’s renal function was satisfactory for the full recommended dose, she was commenced on the lower dose of 150mg once a day as she was over the age of 75.

The obvious advantage of being an oral medication (therefore not requiring skills for administration) and the lack of need for laboratory monitoring and dose alteration have been welcomed both by patients and healthcare workers.^10^ When compared with LMWH, the dabigatran dose regimes of either 150mg or 220mg did not show an increase in bleeding in the RE-NOVATE trial.^1^ Nevertheless, major bleeding remains a potential side effect as with all other VTE prophylaxis agents. Incidence of major bleeding after prophylactic LMWH has been reported as being up to 5.2%.^11^ There have been sporadic case reports of retroperitoneal haematoma resulting in death following administration of LMWH.^12,13^

A case series published in 2012 reported 12 episodes of major bleeding within 2 months in patients newly started on dabigatran and raised potential doubts about the safety of this drug.^14^ The authors identified four major factors (prescriber error, impaired renal function, patient age and complications arising from lack of reversal agent) as the main contributors to bleeding. The site of bleeding can be varied including urethral, oral, rectal, mucosal and subdural sites.

The summary of product characteristics for dabigatran lists active clinically significant bleeding and a lesion or condition at significant risk of major bleeding such as current or recent gastrointestinal ulceration as contraindications.^9^ However, it does not specify a specific time duration and was not a part of the initial NICE guidelines. This issue was raised in the coroner’s report and also accepted by the MHRA as needing further clarification. Although this patient had a previous episode of haematemesis due to a duodenal ulcer two years prior to her hip replacement, she had been asymptomatic since then and no active duodenal ulcers were seen during her upper gastrointestinal endoscopies.

Replacement and correction of the coagulopathy with packed red blood cells, fresh frozen plasma, platelets, vitamin K and tranexamic acid as well as mechanical attempts to limit bleeding were unsuccessful. Although haemofiltration was carried out throughout the patient’s admission as recommended for acute emergency reversal of this agent, it was not possible to reverse by this means in her case. The lack of a confirmed reversal agent proved fatal on this occasion.

There are reports emerging of some reversal success from prothrombin complex concentrate in animal models.^15^ Nevertheless, published in 2011, a randomised study in healthy human subjects showed no influence on the anticoagulant action of dabigatran.^16^ FEIBA® (Factor VIII Inhibitor Bypassing Activity; Baxter, Westlake Village, CA, US) was shown to improve the abnormal thrombin generation in one study from 2013.^17^ However, along with all of the potential antidotes discussed, long-term evidence and further research are still lacking.

## Conclusions

It is hoped that the side effects, as well as the lack of reversal agent, are more widely and seriously appreciated for dabigatran. Further studies are needed to identify possible reversal agents and careful selection of patients deemed suitable to receive this drug for VTE prophylaxis is recommended.
